# Evaluation of Healing Efficacy of Collagen Matrix and Connective Tissue Graft for Root Coverage Using Doppler Flowmetry: A Case Report

**DOI:** 10.7759/cureus.74114

**Published:** 2024-11-20

**Authors:** Shikha Mahapatra, Snophia Suresh, Uma Sudhakar, S. Catherine Jean, Monisha Manikandan

**Affiliations:** 1 Periodontology, Thai Moogambigai Dental College and Hospital, Chennai, IND; 2 Periodontics, Thai Moogambigai Dental College and Hospital, Chennai, IND

**Keywords:** connective tissue graft, coronally advanced flap, gingival recession, mucograft, root coverage

## Abstract

The epitome of periodontal plastic surgical procedures is to achieve coverage of the denuded root surface and flawless esthetics. Connective tissue graft (CTG) along with coronally advanced flap (CAF) is the most frequent approach, which is considered a gold standard remedy. The procurement of CTG requires a second surgical site morbidity. Mucograft and coronally advanced flap procedures are preferable alternative substitutes for root coverage. This case report included two patients with gingival recession. One patient was treated with CAF and CTG; the other was treated with CAF and mucograft. The vascularity was assessed with Doppler flowmetry on days three and five after surgery. The xenogeneic collagen matrix for the management of gingival recession resulted in a comparable amount of vascularization compared to autologous connective tissue graft.

## Introduction

Gingival recession occurs when the gingival margin moves apically beyond the cemento-enamel junction, leading to esthetic concerns as well as increased risk of root caries and sensitivity for patients [1]. Untreated gingival recession defects have a high probability of progression during long-term follow-up [2]. Patients often experience noticeable benefits after root coverage procedures, and there are several plastic surgery techniques available to address gingival recession, with the goal of covering the exposed root surface and achieving optimal esthetics [3].

To obtain complete root coverage, the combination of connective tissue graft (CTG) and coronally advanced flap (CAF) is the most used approach and is considered the gold standard treatment. This CAF+CTG technique has shown the best clinical outcomes for single or multiple gingival recessions. Treatment of gingival recession is still a challenge for the clinician since the wound healing may be compromised by various factors, such as the width of the avascular surface and the limited blood supply, especially after a CAF procedure [4]. Although CTG increases the likelihood of achieving complete root coverage, it involves the risk of additional surgical site morbidity and a higher likelihood of postoperative complications, such as pain and bleeding [5]. Therefore, a newly introduced porcine-derived collagen matrix, Mucograft, serves as an alternative soft tissue replacement material for root coverage in conjunction with the coronally advanced flap procedure [6]. Mucograft consists of two layers: a compact outer layer and an inner spongy collagen layer. The outer layer provides elasticity, making the suturing process easier, while the inner spongy layer helps stabilize the blood clot, thereby promoting regeneration.

Gingival blood flow can be assessed using both invasive and non-invasive methods. Invasive techniques include vital microscopy of the gingival margin, implantation of microspheres into the internal carotid artery in animal studies, infusion of radioisotopes, radio-labeled microspheres, and high-speed cinematography. Doppler flowmetry (DF) is a non-invasive method commonly employed to assess microcirculation in the skin. It operates on the principle of "Doppler Shift," which involves detecting changes in the frequency of light as it is reflected off moving objects, such as red blood cells. In dental practice, Doppler ultrasound is used to evaluate blood flow within the gingival vessels, with colors indicating vascularity: red indicates blood flow toward the ultrasound probe, while blue indicates flow away from it. DF has been applied in dentistry to investigate the effects of periodontal disease, periosteal stimulation, and smoking on gingival blood flow [7].

This case report involves two patients with gingival recession: one was treated with a CAF and CTG, while the other received CAF and Mucograft. Vascularity was assessed using Doppler flowmetry on days three and five.

## Case presentation

**Figure 1:**
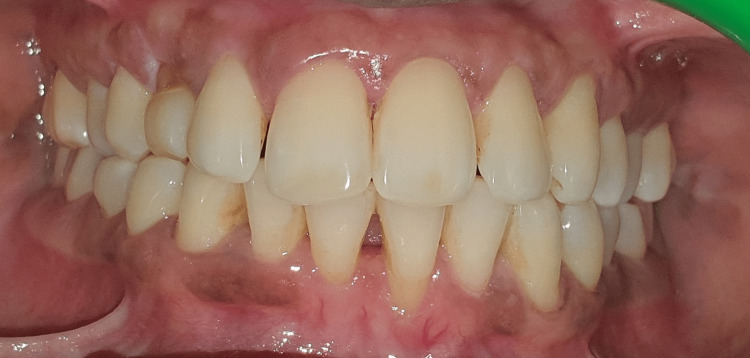
Pre-operative picture for connective tissue graft.

Two systemically healthy patients were selected for the study: Case 1, a 24-year-old woman, and Case 2, a 28-year-old woman, both presenting with Miller’s grade II recession on teeth 31 and 41, respectively (Figure 1). They attended our outpatient clinic, where we discussed the treatment plan and obtained both verbal and written consent from each patient. One week prior to the surgical procedure, they underwent scaling and root planning. Both the patients had 3 mm of keratinized tissue located apical to the gingival recession. A coronally advanced flap procedure with a connective tissue graft was planned on case 1 and Mucograft on case 2 for root coverage.

**Figure 2:**
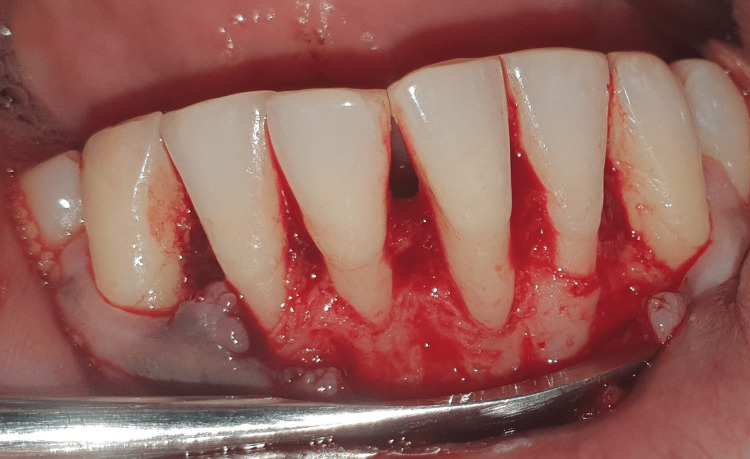
Flap reflected in the lower anterior region.

**Figure 3:**
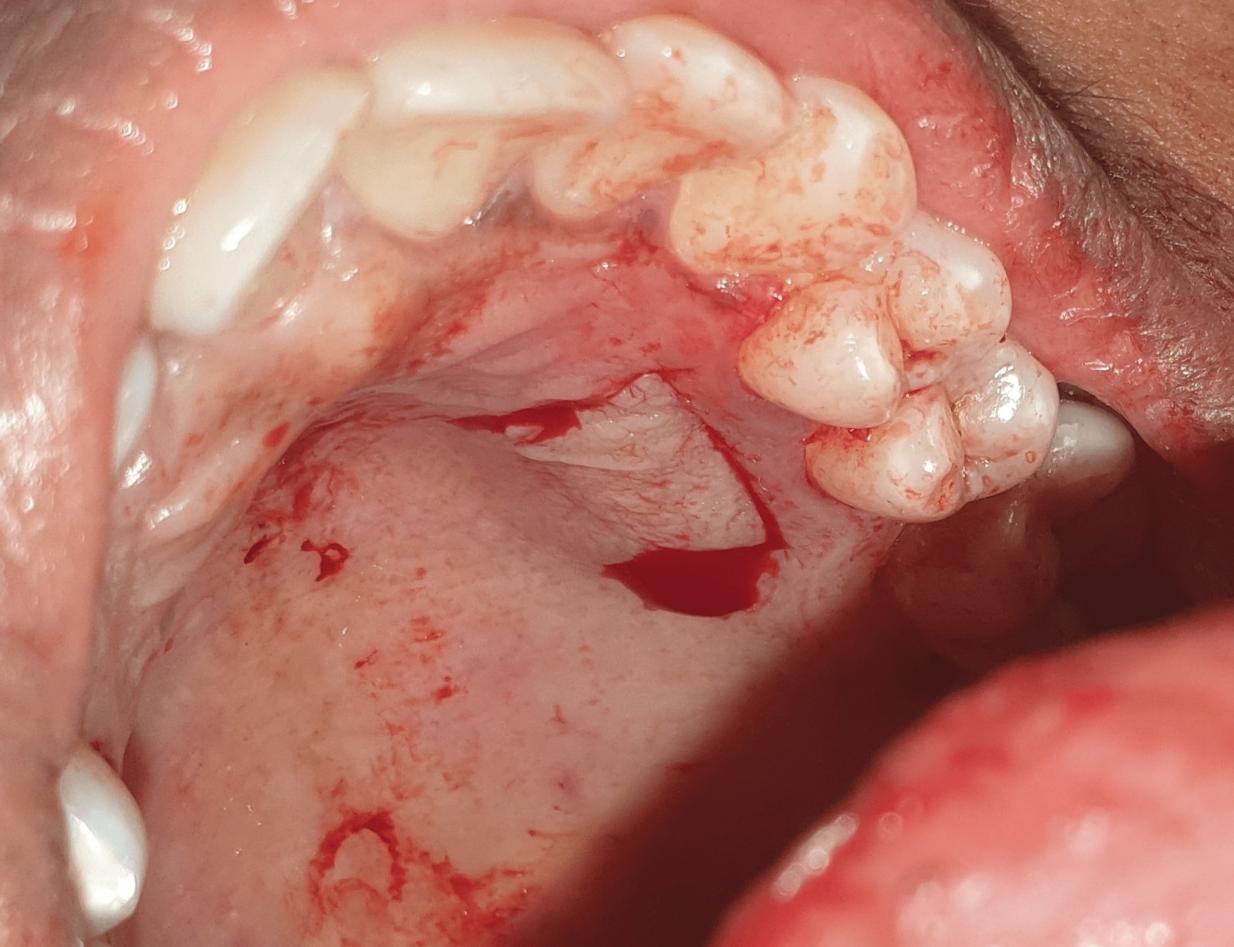
Partial thickness flap reflected in the palate.

**Figure 4:**
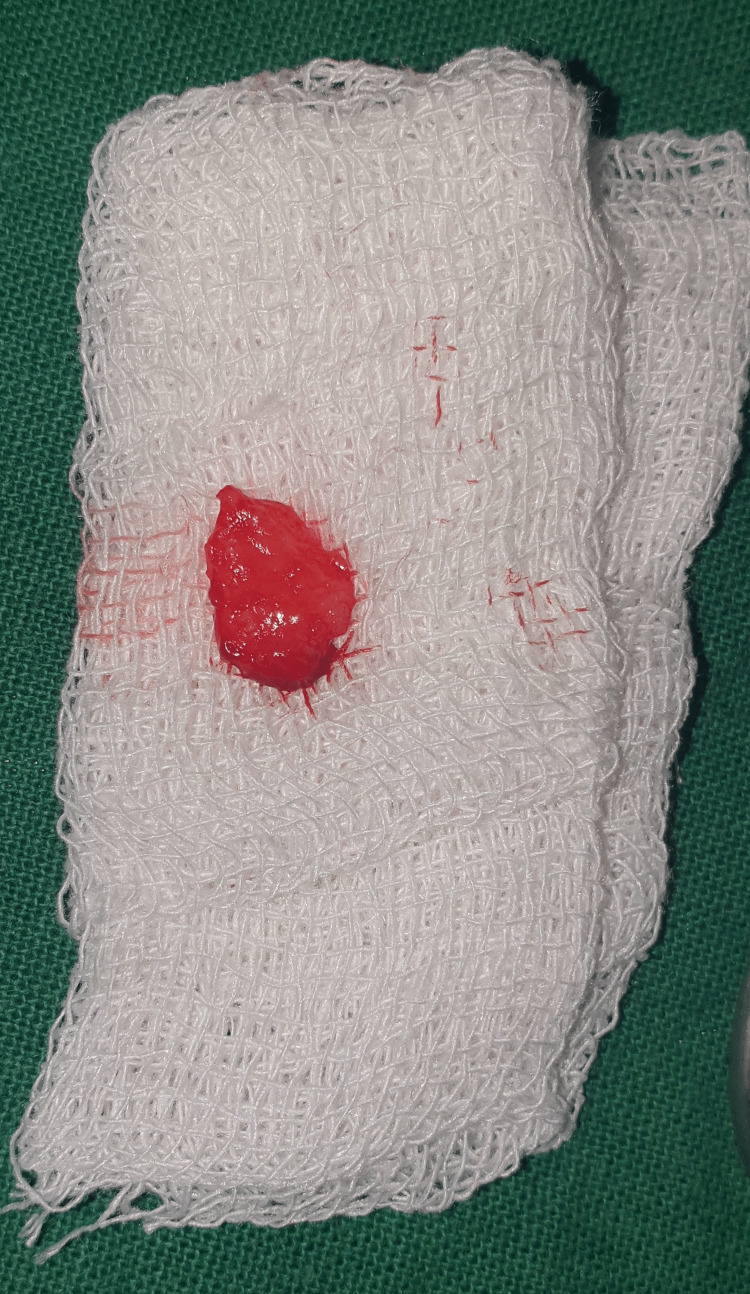
Harvested connective tissue graft.

**Figure 5:**
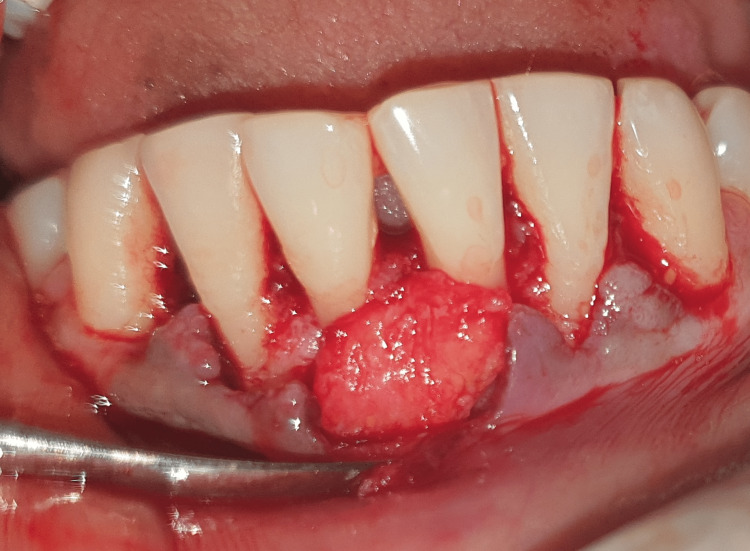
Connective tissue graft fixed at the recipient site.

**Figure 6:**
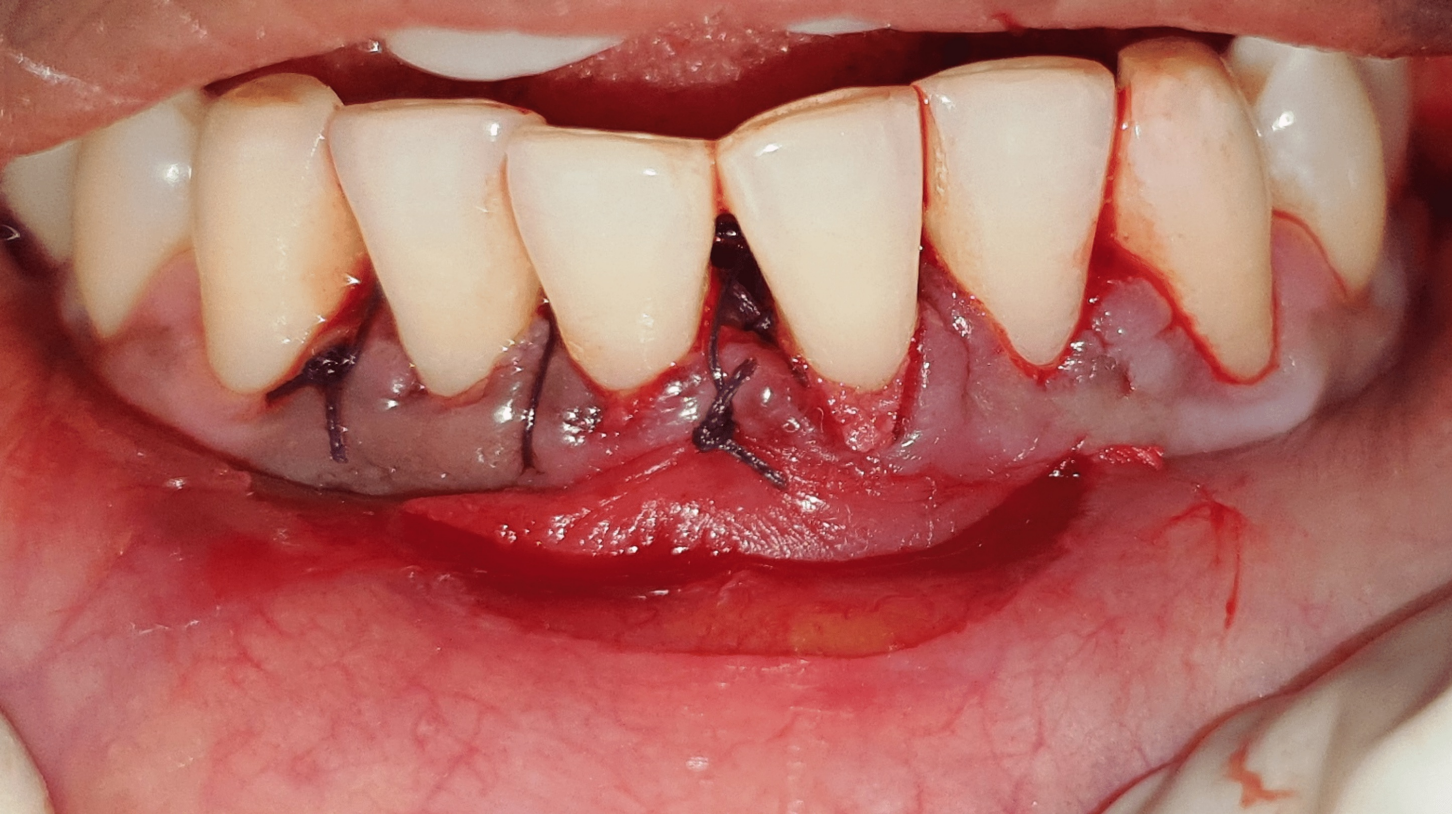
Recipient site at the end of surgery.

**Figure 7:**
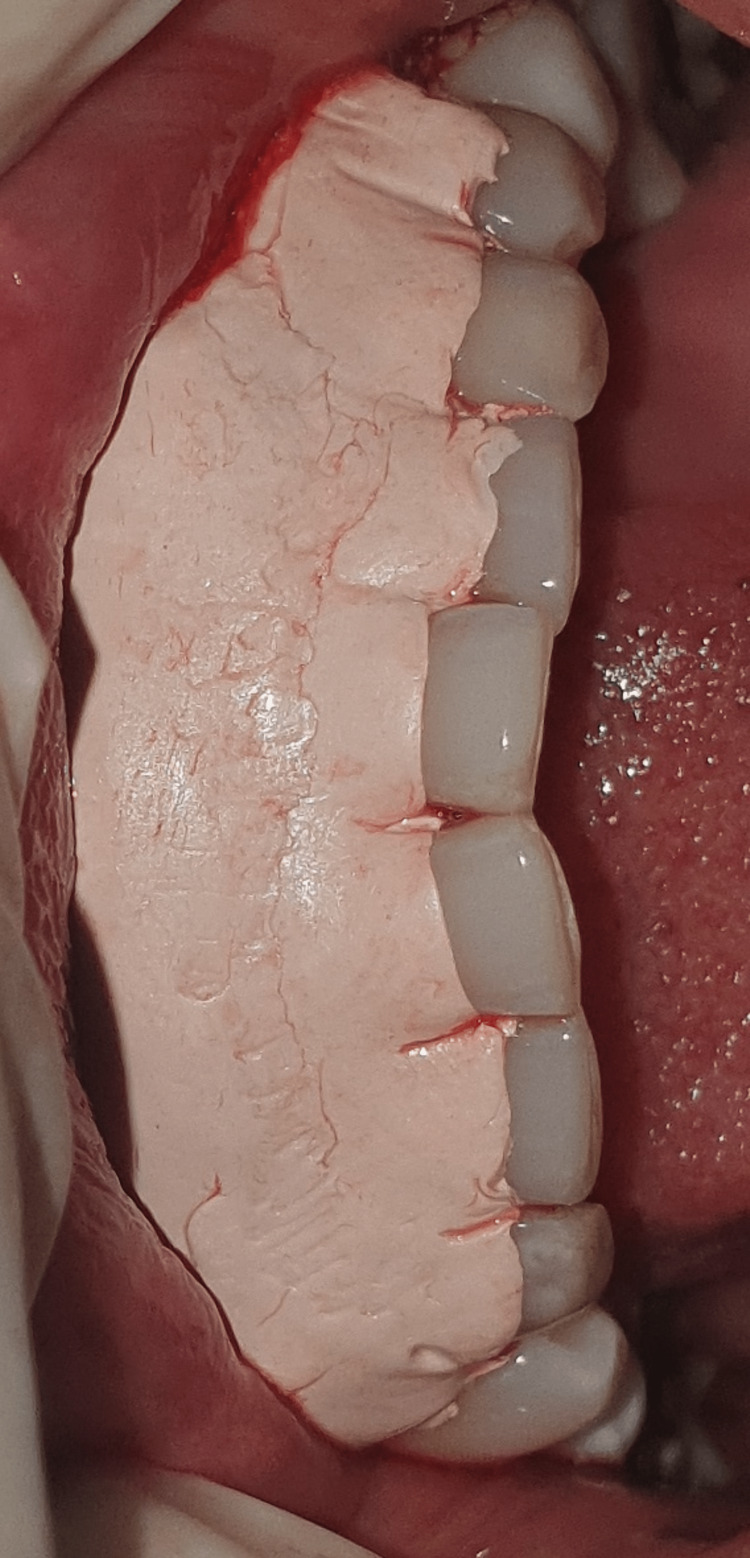
Periodontal dressing placed after CAF and CTG procedure. CAF: coronally advanced flap, CTG: connective tissue graft.

**Figure 8:**
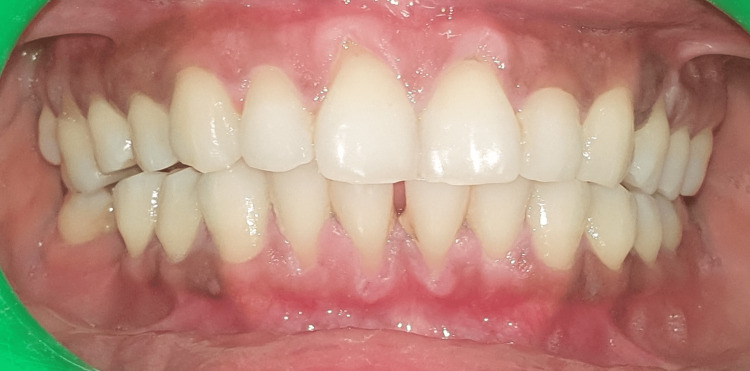
Pre-operative picture for CAF and Mucograft procedure. CAF: coronally advanced flap.

**Figure 9:**
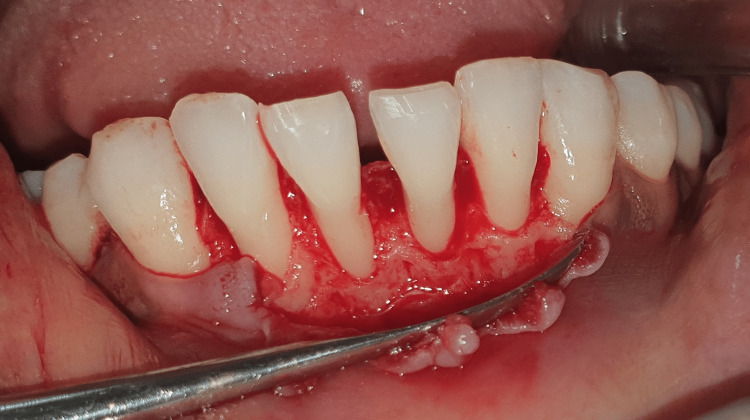
Flap reflected in the lower anterior region.

**Figure 10:**
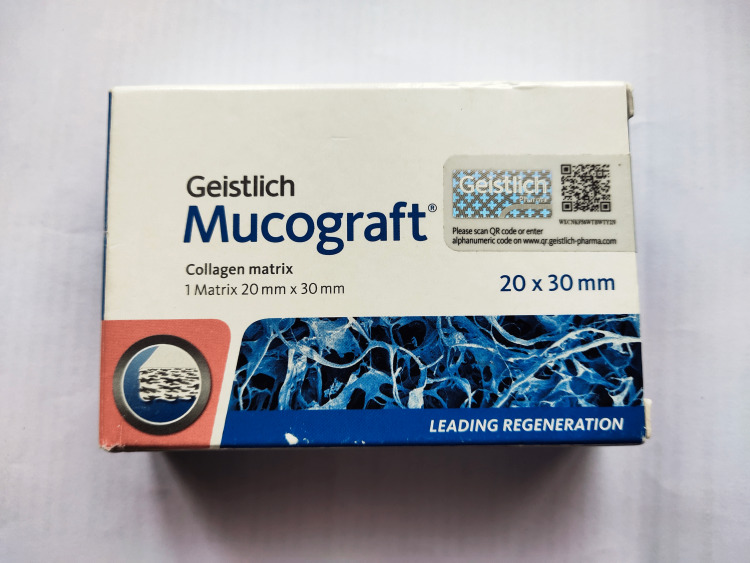
Collagen Matrix Mucograft used for the procedure.

**Figure 11:**
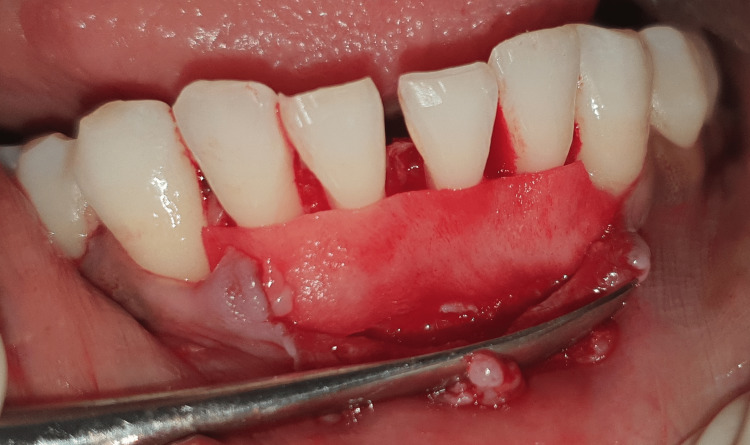
Mucograft placed at the site.

**Figure 12:**
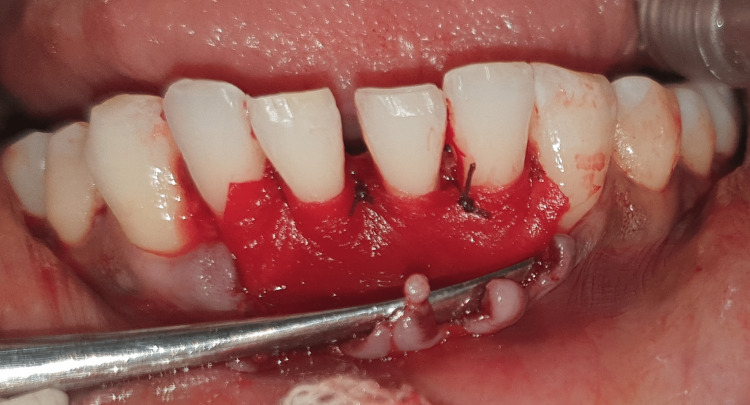
Mucograft has been sutured.

**Figure 13:**
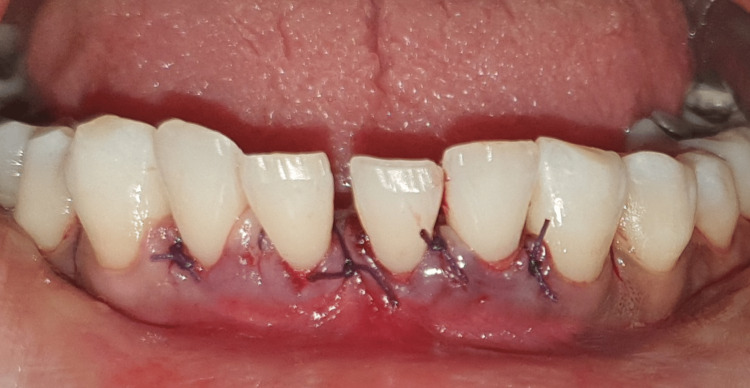
Flaps approximated and sutured.

**Figure 14:**
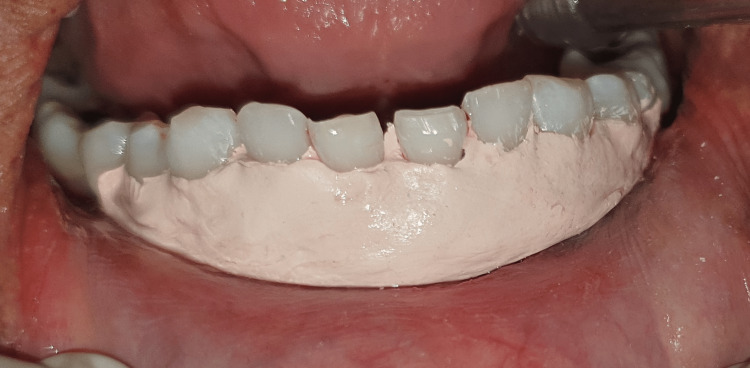
Periodontal dressing placed after CAF and Mucograft procedure. CAF: coronally advanced flap.

All surgeries took place in a single session. Patients were given local anesthesia using 2% lignocaine containing 1:80,000 epinephrine for the procedure. The coronally advanced flap technique involved making a horizontal intrasulcular incision to raise a full-thickness gingival flap up to the mucogingival junction, and a horizontal releasing incision was placed in the periosteum at the base of the flap to ensure tension-free displacement of the flap [8]. A connective tissue graft was obtained from the palate using a trap door incision and secured with a sling suture in case 1 (Figures 2-7). In the trap door technique, a 15-no. blade was used to give one horizontal and two vertical incisions to raise the flap, and the connective tissue was dissected at its base using a horizontal incision and the primary flap was repositioned. The donor connective tissue was sutured to the recipient bed using a 4-0 Vicryl suture (Ethicon, Raritan, USA), and the flap was coronally advanced and sutured. In case 2 (Figure 8), the Mucograft was shaped and sized appropriately, ensuring that the compact side faced the underlying tissue while the spongy side was oriented outward. The graft was positioned over the defect and extended apically beyond the base of the recession defect. The graft was stabilized with a 4-0 Vicryl suture in case 2 (Figures 9-14). A periodontal dressing (Coe-Pak) was applied over the surgical area to prevent friction. Systemic antibiotics and analgesics were prescribed for seven days after surgery. Sutures were removed after ten days, and patients were scheduled for a follow-up appointment one month postoperatively.

Ultrasound Doppler flowmetry

**Figure 15:**
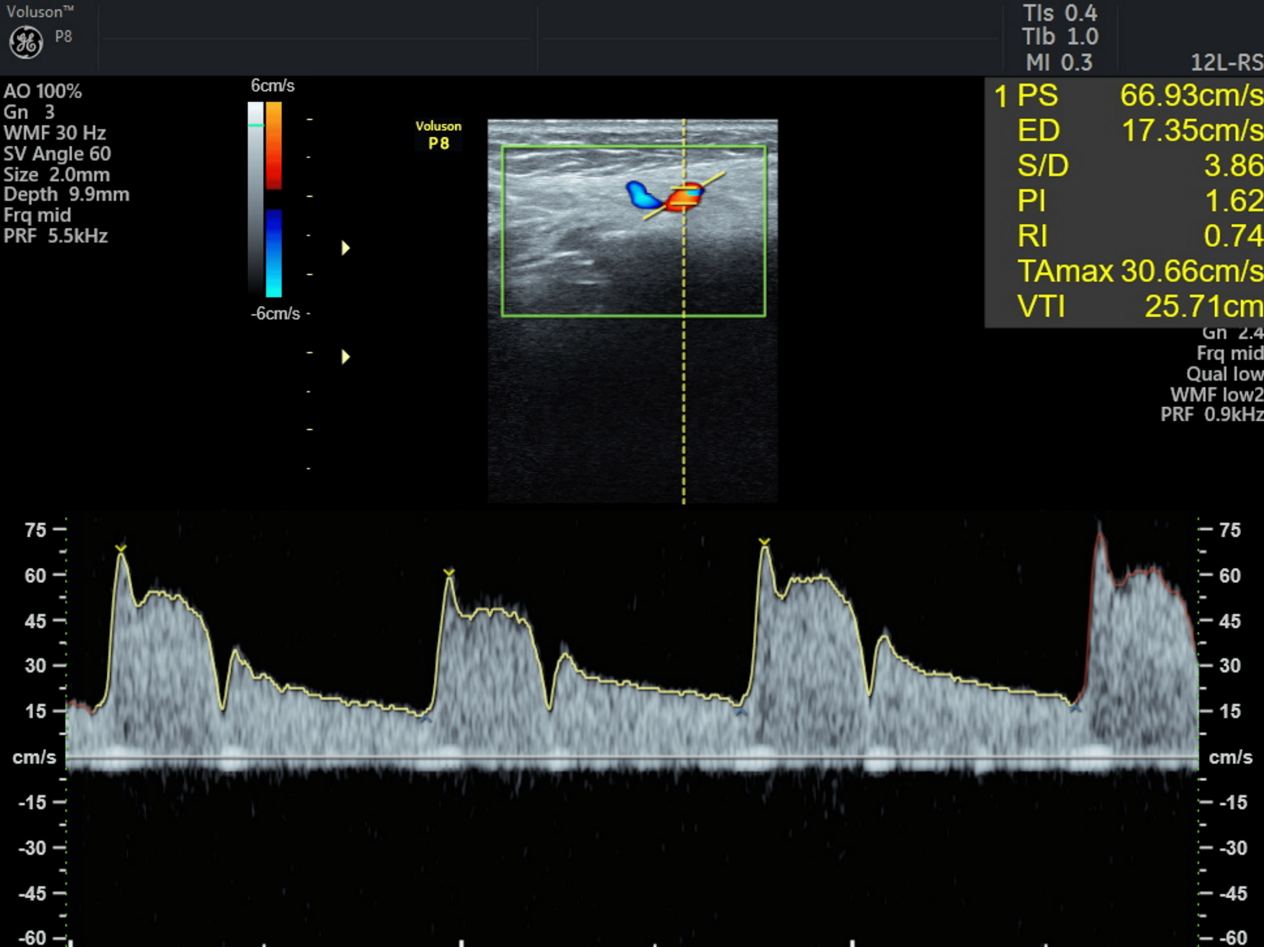
Ultrasound Doppler flowmetry of CAF + CTG on the third day. CAF: coronally advanced flap, CTG: connective tissue graft, PS: peak systolic velocity, ED: end-diastolic velocity, PI: pulsatility index, RI: resistive index, VTI: velocity time integral.

**Figure 16:**
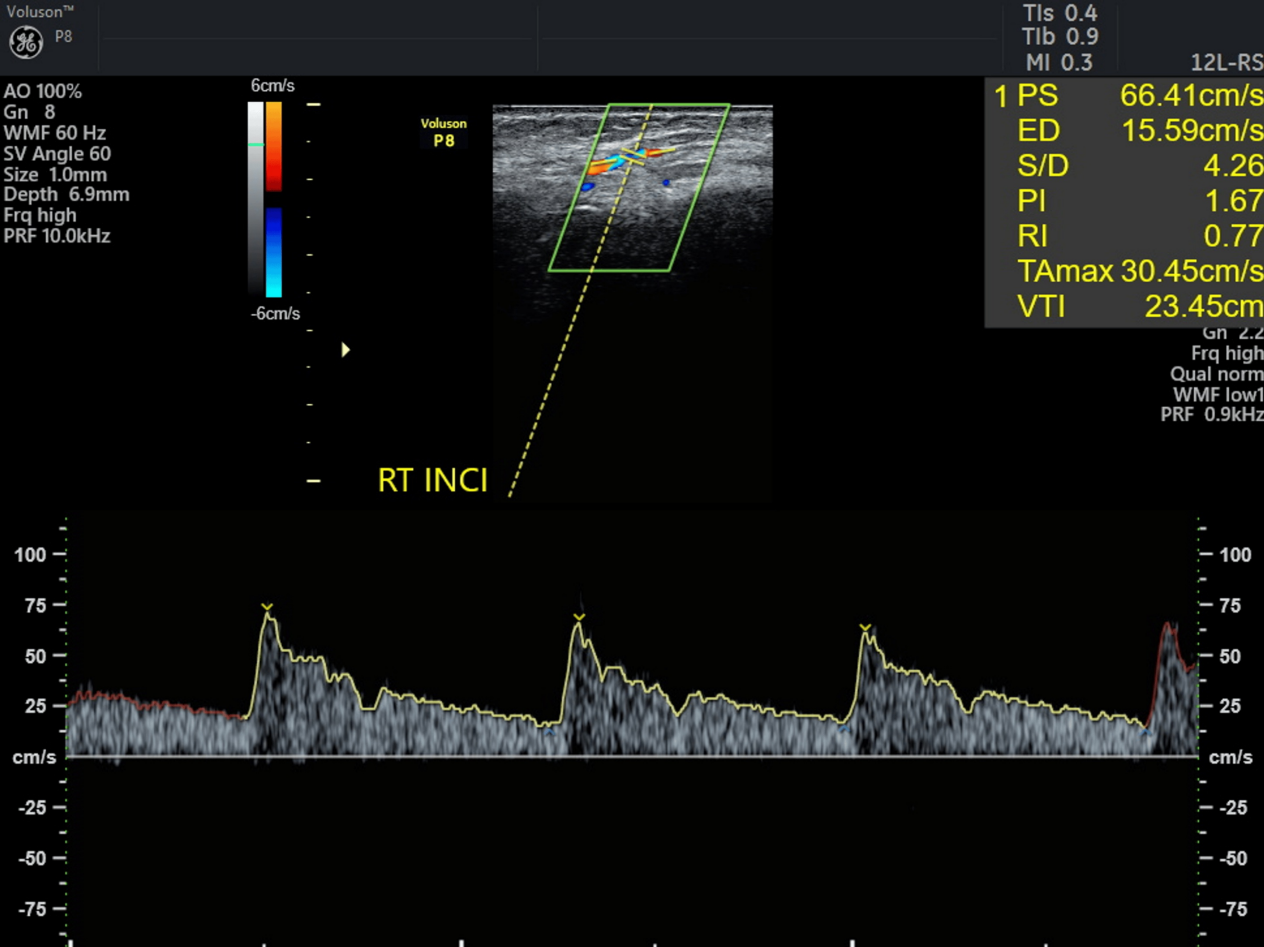
Ultrasound Doppler flowmetry of CAF + Mucograft on the third day. CAF: coronally advanced flap, CTG: connective tissue graft, PS: peak systolic velocity, ED: end-diastolic velocity, PI: pulsatility index, RI: resistive index, VTI: velocity time integral, RT INCI: right incisor.

**Figure 17:**
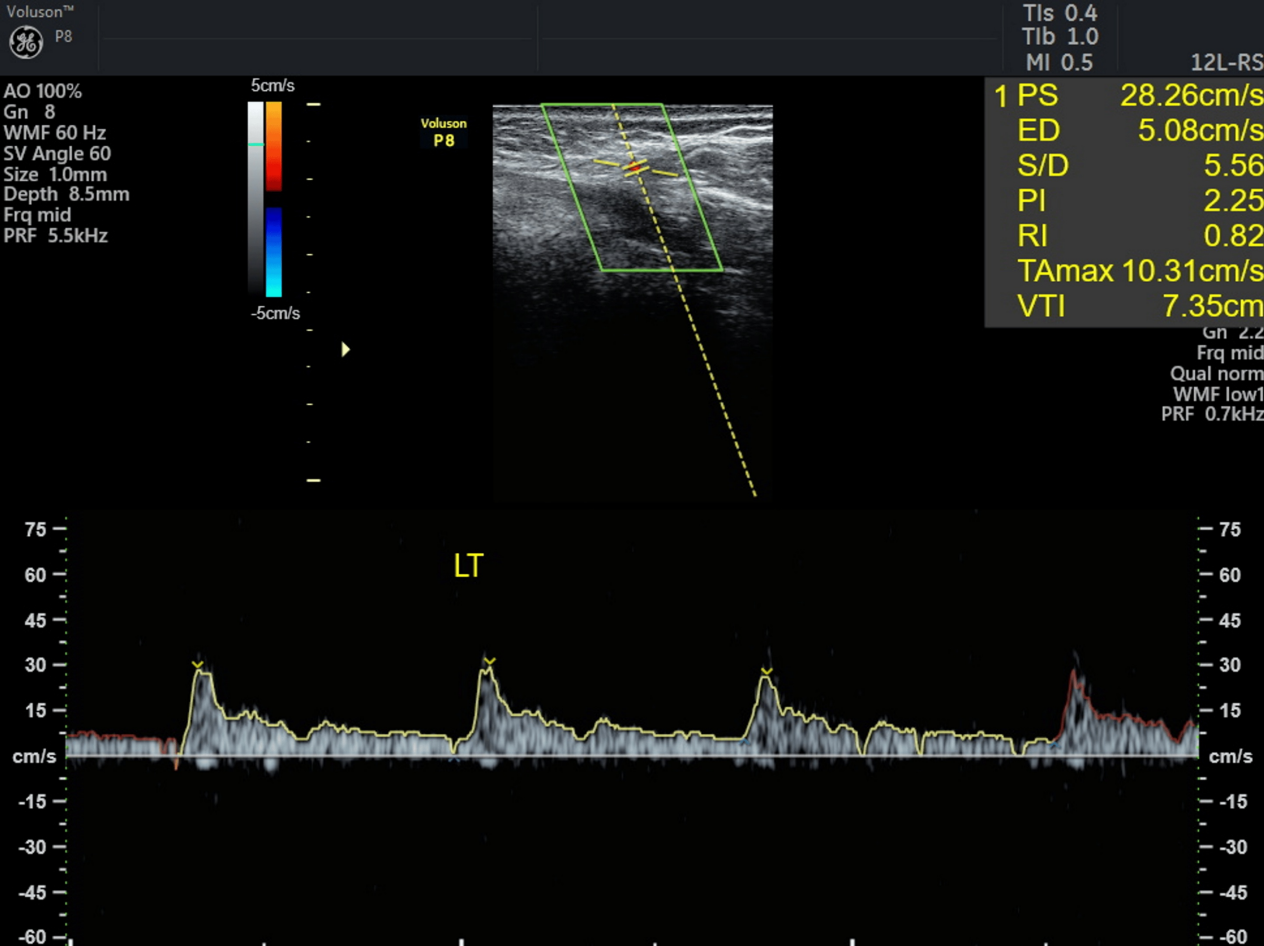
Ultrasound Doppler flowmetry of CAF + CTG on the fifth day. CAF: coronally advanced flap, CTG: connective tissue graft, PS: peak systolic velocity, ED: end-diastolic velocity, PI: pulsatility index, RI: resistive index, VTI: velocity time integral, LT: left.

**Figure 18:**
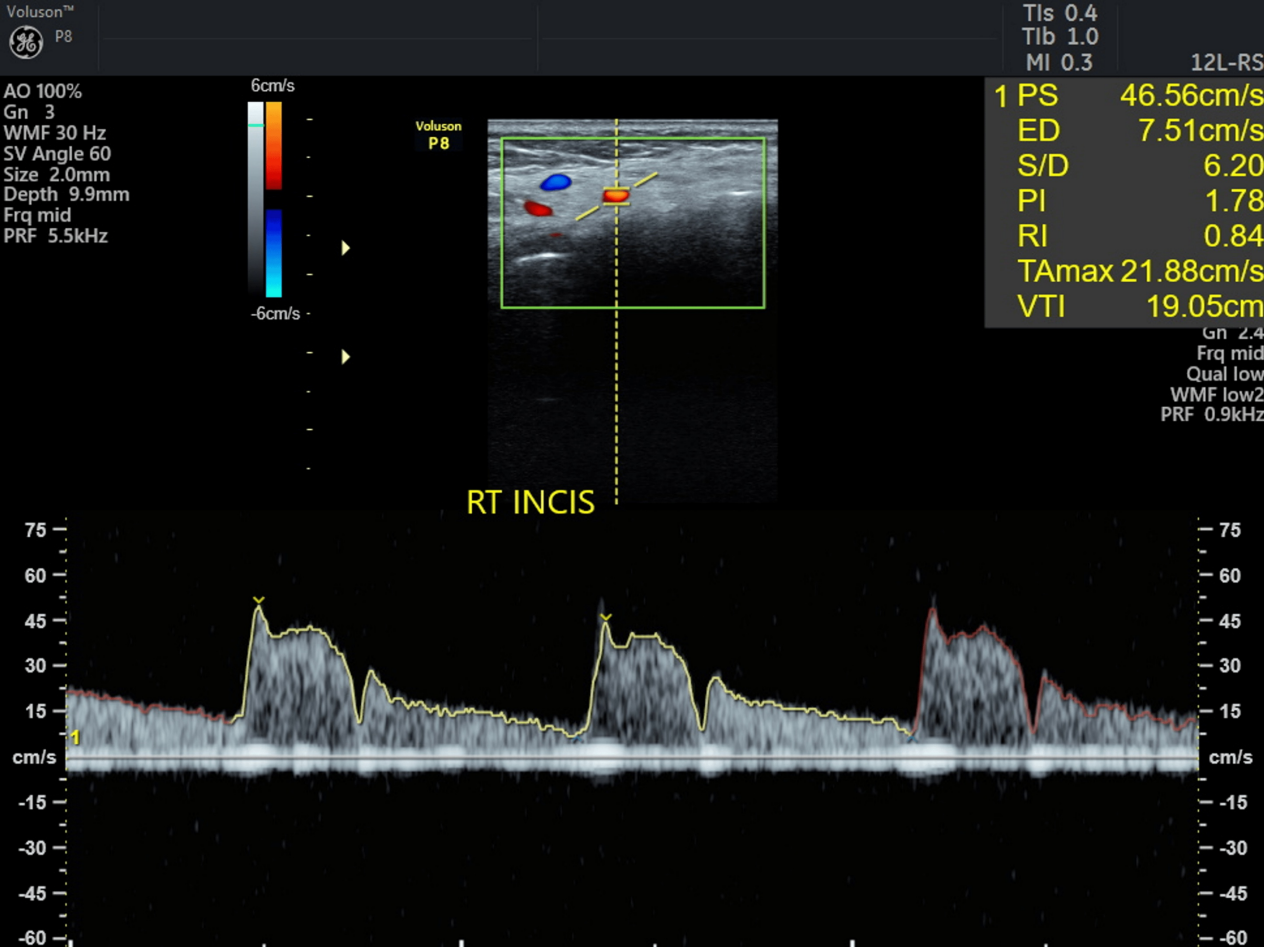
Ultrasound Doppler flowmetry of CAF + Mucograft on the fifth day. CAF: coronally advanced flap, CTG: connective tissue graft, PS: peak systolic velocity, ED: end-diastolic velocity, PI: pulsatility index, RI: resistive index, VTI: velocity time integral, RT INCI: right incisor.

Following the surgery, blood flow during the immediate healing period was measured at both sites using ultrasound Doppler flowmetry on the third- and fifth-days post-surgery (Figures 15-18). The periodontal dressing was removed to allow the Doppler probe to contact the targeted mucosal site for accurate detection of red blood cell movement. Color Doppler ultrasound imaging was performed using an ACUSON X300™ ultrasound system (Siemens GE, Mountain View, CA, USA) featuring 4D transducer technology. A linear transducer probe (VF10-5) (Siemens GE, Mountain View, CA, USA), operating at a frequency of 10 MHz, was used for the procedure. The transducer was placed over a gloved finger in a coronal plane, with ultrasound gel applied to ensure optimal contact. The water-filled glove finger served as a medium for sound waves, improving the clarity and detail of the images.

Results

Doppler ultrasound is frequently used to evaluate blood flow within gingival vessels, with images displayed in red or blue to indicate vascularity: red indicates blood flow toward the ultrasound probe, while blue indicates flow away from it. Neovascularization was noted in 31 and 41 regions for both patients on the third and the fifth day. Pulsatility index (PI) and resistive index (RI) are used to assess the hemodynamic status of a patient. The ultrasound's resistive index (RI) is a calculated blood flow parameter derived from the mean Doppler frequency shifts during a defined cardiac cycle. RI is calculated by subtracting the end-diastolic velocity (ED) from the peak systolic velocity (PS) and then dividing it by the PS value. PI is defined as the peak systolic flow velocity (PS) minus the diastolic velocity (ED) divided by the mean velocity. PI is a calculated flow parameter that is derived from the maximum, minimum, and mean Doppler frequency shifts during a cardiac cycle. A normal high-resistant flow pattern was observed on the third day in cases 1 and 2 with RI of 0.74 and 0.77 and PI of 1.67 and 1.62, respectively, indicating similar vascular flow on the third day. On the fifth day, an increase in blood flow was observed with RI of 0.82 and 0.84 and PI of 2.25 and 1.78 in both cases, with similar blood flow patterns. TAMAX, or time-averaged maximum velocity, is a time integral of the fastest velocity layers in an ultrasound device. Velocity time integral (VTI) measures how far blood travels during the time period. 

## Discussion

This case report is the first to evaluate vascularity following the use of connective tissue grafts and mucografts. The innovative non-invasive method of ultrasound Doppler flowmetry was employed to assess vascularization after periodontal surgery. While various techniques, such as impedance plethysmography and microsphere implantation, have been used to study gingival blood flow, most of these methods are invasive or not suitable for human subjects. In most studies, laser Doppler flowmetry is the primary method used to assess blood flow. This technique has been utilized to measure pulpal blood flow, perfusion after flap surgery, and blood flow in both gingival health and inflammation. However, we opted for Doppler sonography due to its added benefits, including the ability to compare blood flow changes between different sites within the same patient as well as across different patients. Furthermore, Doppler sonography has not been previously explored, particularly in the context of periodontal plastic surgery. The vascular supply in the lower anterior region, corresponding to the surgical sites in both groups, was clearly observed in the treated cases.

Guiha et al. reported that in cases of connective tissue graft (CTG) combined with coronally advanced flap (CAF), vascularization originated from both the periodontal plexus and the overlying flap, resulting in an effective blood supply to the graft two weeks after the surgical procedure [9]. The spongy scaffold in Mucograft facilitates the organization of the blood clot. It promotes the formation of new blood vessels, as well as tissue integration and the ingrowth of soft tissue cells. Aroca et al. noted that Mucograft, as a 3D scaffold, allows for the ingrowth of blood vessels and fibroblasts from surrounding tissues, and this material has been explored as an alternative to free gingival grafts [10].

A recent trial by Cairo et al. [11] offers additional insights, suggesting that the thickness of the flap margin may influence the short-term benefits of adding connective tissue graft (CTG) to a coronally advanced flap. Consequently, the adjunctive use of CMX or CTG with coronally advanced flaps may provide advantages at recession sites with thinner marginal tissues.

The latest generation of collagen matrices, which offer enhanced volumetric stability during the healing period, is important for interpreting the current results. Both techniques showed satisfactory clinical outcomes in this case report. The adjunctive use of xenogeneic collagen matrices with coronally advanced flaps for managing gingival recessions results in a level of vascularization comparable to that achieved with autologous connective tissue grafts. While connective tissue grafts are regarded as the gold standard, they have drawbacks, such as the need for a second surgical site and potential discomfort for the patient.

## Conclusions

Both the autogenous connective tissue graft and the xenogenic collagen matrix (Mucograft) demonstrated no differences in the revascularization process. The results for vascularization were comparable for both grafts, indicating that Mucograft can serve as an alternative to connective tissue grafts for root coverage. Future multicenter randomized clinical trials involving larger patient populations are necessary to validate these findings.
